# Elevated hydrogen peroxide and decreased catalase and glutathione peroxidase protection are associated with aging sarcopenia

**DOI:** 10.1186/1471-2318-13-104

**Published:** 2013-10-07

**Authors:** Melanie J Sullivan-Gunn, Paul A Lewandowski

**Affiliations:** 1 School of Biomedical and Health Sciences, Victoria University, Melbourne, Australia; 2 School of Medicine, Deakin University, Geelong 3217, Vic, Australia

**Keywords:** Sarcopenia, NADPH oxidase, Superoxide, Hydrogen Peroxide, Antioxidants

## Abstract

**Background:**

Sarcopenia is the progressive loss of skeletal muscle that contributes to the decline in physical function during aging. A higher level of oxidative stress has been implicated in aging sarcopenia. The current study aims to determine if the higher level of oxidative stress is a result of increased superoxide (O_2_‾) production by the NADPH oxidase (NOX) enzyme or decrease in endogenous antioxidant enzyme protection.

**Methods:**

Female Balb/c mice were assigned to 4 age groups; 6, 12, 18 and 24 months. Body weight and animal survival rates were recorded over the course of the study. Skeletal muscle tissues were collected and used to measure NOX subunit mRNA, O_2_‾ levels and antioxidant enzymes.

**Results:**

Key subunit components of NOX expression were elevated in skeletal muscle at 18 months, when sarcopenia was first evident. Increased superoxide dismutase 1 (SOD1) activity suggests an increase in O_2_‾ dismutation and this was further supported by elevated levels of hydrogen peroxide (H_2_O_2_) and decline in catalase and glutathione peroxidase (GPx) antioxidant protection in skeletal muscle at this time. NOX expression was also higher in skeletal muscle at 24 months, however this was coupled with elevated levels of O_2_‾ and a decline in SOD1 activity, compared to 6 and 12 months but was not associated with further loss of muscle mass.

**Conclusions:**

While the source of ROS in sarcopenic muscle remains unknown, this study provides evidence that the NOX enzyme could be involved in ROS production by regulating superoxide in ageing muscles. This study also suggests that H_2_O_2_ is the key ROS in the onset of sarcopenia and that the decline in antioxidant protection by catalase and GPx is indicative of antioxidant dysfunction and may therefore be a major contributing factor in the development or onset of sarcopenia. Furthermore, the changes in ROS and antioxidant activity after sarcopenia was first evident gives some evidence for a compensatory mechanism, in response to insult, in order to maintain muscle integrity.

## Background

One of the most dramatic phenotypic changes during aging is sarcopenia [1,2]. Sarcopenia is characterised by a significant loss of skeletal muscle mass [3] at a rate of 5% per decade, starting in the fourth decade [4] with a prevalence of 5-13% in people 60-70 years of age and 11-50% of people over 80 years [5]. Consequently, the progressive age-associated loss of muscle mass leads to significant weakness, frailty and decreased quality of life [6]. It has recently been reported that sarcopenia causes significant frailty in the elderly that any stressor leads to a poor outcome, disability and mortality [5] despite age, clinical and functional variability [7].

The free radical theory of aging postulates that endogenous reactive oxygen species (ROS) cause progressive cellular damage and that incomplete repair leads to an accumulation over time [8]. ROS are generated in skeletal muscle as by-products of cellular metabolism, but are capable of exceeding beyond normal cellular levels, during exercise [1] and in disease [9-11] and the ability of cells to actively detoxify ROS becomes less efficient over time [12].

The knowledge that the NADPH oxidase (NOX) enzyme is a primary superoxide (O_2_‾) producing system [13] has brought about much speculation for its role in skeletal muscle. The NOX catalytic subunit NOX2 [9], associated subunit components, p22^*phox*^, p40^*phox*^, p47^*phox*^ and p67^*phox*^[14], GTP binding protein Rac1 [15] and the NOX2 homologue NOX4, have been found in skeletal muscle tissue [16]. Although limited research exists, aging studies have demonstrated an age-associated increase in ROS as a consequence of NOX2 enzyme up regulation [11] and activity [17], and decreases in endothelial dysfunction and vascular aging have been reported in NOX2 deficient mice [18,19]. Overexpression of NOX4 has been proposed to lead to cellular senescence in fibroblasts [20] and degenerative dysfunction associated with aging [21]. Furthermore, NOX has been identified as the key source of O_2_‾ that contributes to cardiovascular aging [11] and other age-associated conditions such as diabetes [22], arthritis [23], cataracts [24], Alzheimer’s disease [25] and motorneuron dysfunction [10].

The high metabolic activity of skeletal muscle makes it particularly vulnerable to excess ROS production and oxidative stress [26]. Antioxidant enzymes are therefore important in skeletal muscle and contrasting studies have propose both a compensatory increase in antioxidant activity [27] as well as evidence to suggest antioxidant dysfunction [12] during aging. Superoxide dismutase (SOD) is responsible for the dismutation of O_2_‾ and elimination of cellular oxidative stress. The absence of the cytosolic SOD1 isoform is known to significantly decrease lifespan [28] and more recently, high levels of oxidative stress and accelerated sarcopenia due to O_2_‾ induced neuromuscular degeneration and mitochondrial dysfunction has been shown in SOD1 deficient mice [29-31].

Oxidative stress is recognised as a major contributor to aging and age-associated disease [10,29,32] and evidence suggests its involvement in the development of sarcopenia [1]. However, the key contributors to oxidative stress in skeletal muscle during aging and changes within these systems throughout the lifespan, remains undefined. Therefore, this study aims to investigate the NOX and antioxidant enzyme systems in aging skeletal muscle. In particular, this study aims to determine changes in the NOX proteins; NOX2, NOX4, p22^*phox*^, p40^*phox*^, p47^*phox*^, p67^*phox*^ and Rac1, O_2_‾ and H_2_O_2_ levels and associated antioxidants; SOD isoforms, catalase and GPx, in skeletal muscle during aging.

## Methods

### Animal model of aging sarcopenia

All animal procedures carried out in this study were approved by the Victoria University Animal Ethics Committee (AEETH 14/05). Female Balb/c mice were assigned to 4 age groups; 6, 12, 18 and 24 months of age and maintained under controlled environmental conditions; 12 hour light/dark cycle, 21 ± 2C, 30% humidity, in conventional cages with *ad libitum* access to standard chow and water. Body weight measurements and animal survival rates were recorded over the course of the study. Skeletal muscle tissues were collected from 6 (*n =* 16), 12 (*n =* 18), 18 (*n =* 12) and 24 (*n =* 12) month old mice and immediately snap frozen in liquid nitrogen and stored at -80°C for later use.

### Detection of O_2_‾ by DHE fluorescence staining

Skeletal muscle O_2_‾ was measured by histological Dihydroethidium (DHE) fluorescence examination. DHE is cell permeable and reacts with O_2_‾ converting DHE into ethidium fluorescence [33]. DHE (5 μm/L) was applied to quadriceps cryosections (5 μm) and incubated in a light protected oven at 37°C for 30 minutes. The DHE was washed from the sections with PBS and fluorescence was assessed using Axiocam HBO 50/AC fluorescence microscopy (Zeiss, Germany). The intensity of ethidium fluorescence detection of O_2_‾ was measured with MCID imaging software (Imaging Research Inc. Australia) with excitation at 480 nm and emission at 560 nm. Values represent the area of fluorescence, above a specified threshold, expressed as a percentage of the total area of the muscle cross-section [34].

### Reverse transcription-real-time PCR

RNA was extracted from frozen quadriceps muscle using Tri Reagent (Molecular Research Centre, USA), according to the manufacturer’s protocol. Total RNA concentration was determined spectrophotometrically at 260 nm. First-strand cDNA was generated from 1 μg RNA using AMV RT (Promega, Australia). Pre-designed TaqMan Gene Expression Assays (Applied Biosystems, USA) were used containing specific primers and probes for the gene of interest. Real-time PCR was performed using Applied Biosystems 7500 detection system following manufacturer’s instructions (Applied Biosystems, USA) and PCR reactions were performed using TaqMan Gene Expression Master Mix (Applied Biosystems, USA). To compensate for variations in input RNA amounts and efficiency of reverse transcription, 18 s ribosomal mRNA was quantified and all results were normalised to these values. Fluorescent emission data was captured and mRNA levels were analysed using the critical threshold value (*C*_T_) [35].

### Protein electrophoresis and western blotting

Protein was extracted from frozen quadriceps muscle homogenized in ice-cold radio- immunoprecipitation assay (RIPA) buffer containing Tris HCl (50 mmol/L; pH 7.4), NaCl (150 mmol/L), NP-40 (1%), sodium deoxycholate (0.5%) and SDS (0.1%) and centrifuged at 13,000 × g for 15 minutes at 4°C, to remove insoluble material. The protein concentration was determined by the Bradford method (Bio-Rad) and equal amounts of protein were separated by SDS-PAGE and transferred to polyvinylidene difluoride membranes. The membranes were blocked for 2 hours at room temperature in Tris-buffered saline containing Tris-HCl (20 mM; pH 7.6) NaCl (137 mM) and Tween 20 (0.1%) with 5% BSA and probed with primary antibodies for either NOX2 (gp91^*phox*^), p40^*phox*^, p67^*phox*^ or GAPDH (1:200; SantaCruz Biotechnology) overnight at 4°C. Antibody binding was detected using horseradish peroxidase conjugated secondary antibody (1:50,000; SantaCruz Biotechnology). The protein bands were detected by SuperSignal West Dura chemiluminescence reagents (Thermo Scientific). The LAS 4000 Imaging System (Fujifilm Life Science, USA) was used to visualize protein bands and densitometry was performed with MultiGauge software (Fujifilm Life Science, USA). To compensate for variation in protein loading the relative density of immunoreactive bands were normalized to the density of the corresponding bands for GAPDH.

### Antioxidant enzyme activity and hydrogen peroxide assays

Spectrophotometric assay kits were used to measure SOD (Cayman-706002), catalase (Cayman-707002) and GPx (Cayman-703102) activity and H_2_O_2_ (Cayman-600050) levels, in muscle homogenates. Frozen muscle pieces (100 mg) were placed in ice-cold HEPES buffer (20 mM) containing EGTA (1 mM), mannitol (210 mM) and sucrose (70 mM) and adjusted to a pH of 7.2 (10 ml/g). Muscle aliquots were homogenised in buffer, using a glass on glass homogeniser, and centrifuged at 1,500 × g for 5 min at 4°C to remove insoluble connective tissue. For the detection of SOD1 and SOD2, cytosolic and mitochondrial fractions were separated. The supernatant was centrifuged at 10,000 × g for 5 min at 4°C, and the resulting supernatant, containing the cytosolic fraction, was collected for SOD1 enzyme analysis. The remaining pellet containing the mitochondrial fraction was resuspended and homogenised in ice cold HEPES buffer (20 mM) for SOD2 enzyme analysis. SOD1 and SOD2 activity was measured in cytosolic and mitochondrial muscle fractions, respectively. The amount of enzyme activity and H_2_O_2_ levels were calculated and standardised for protein using the Bradford method (Bio-Rad).

### Statistical analysis

Statistical analysis was performed using SPSS statistical package (version 15.0). Results were expressed as mean ± SEM. Differences were determined by one-way ANOVA with Tukey HSD as posthoc and results were considered statistically significant if *p* values ≤0.05.

## Results

### Establishment of murine model of aging sarcopenia

**Figure 1:**
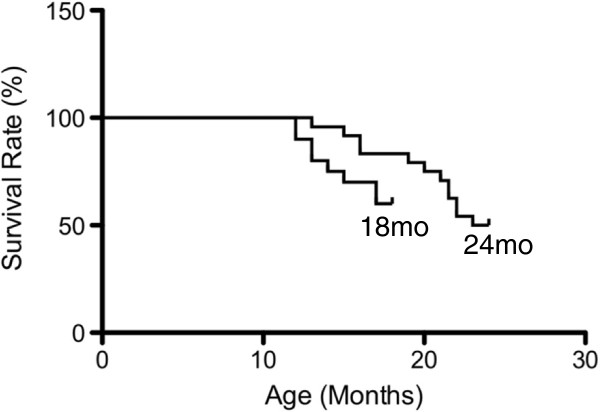
**Animal survival rates in the 18 and 24 month old age groups, expressed as a percentage of live animals for each group.** Female BALB/c mice purchased at 9 months of age were assigned to the 18 month old group (*n =* 20) and 24 month old group (*n =* 24). Animal survival declined from approximately 14 months of age in both groups and continued to decline thereafter. A 60% decline in animal survival was observed in the 18 month old group (18 m/o), while a 50% decline in survival rate was observed the 24 month old group (24 m/o).

Survival rates of female Balb/c mice began to decline at approximately 14 months of age with a 60% survival rate in the 18 month group and a 50% survival rate in the 24 month group (Figure 1). Skeletal muscle mass and muscle mass as ratio to body weight increased at 12 months of age compared to 6 months of age (*p* < 0.001, Table 1) and decreased at 18 (*p <* 0.001) and 24 (*p* < 0.001) months of age compared to 12 months (Table 1).

**Table 1:** Body weight and quadriceps muscle weights

	**Age (months)**			
	**6 (*n =*** **16)**	**12 (*n =*** **18)**	**18 (*n =*** **12)**	**24 (*n =*** **12)**
Body weight (g)	30 ± 1.8	27 ± 2.2	28 ± 1.3	28 ± 1.3
Quadriceps weight (g)	0.15 ± 0.02	0.18 ± 0.01^acd^	0.15 ± 0.02	0.16 ± 0.01
Body weight/quadriceps weight ratio	5.08 ± 0.59	6.15 ± 1.72^acd^	5.45 ± 0.38	5.55 ± 0.52

The values represent the mean ± SEM.

Statistically significant differences (P < 0.05) between groups are indicated by lower case letters, where ^a^6 m/o, ^c^18 m/o and ^d^24 m/o.

### O_2_‾ Levels in skeletal muscle of aging mice

**Figure 2:**
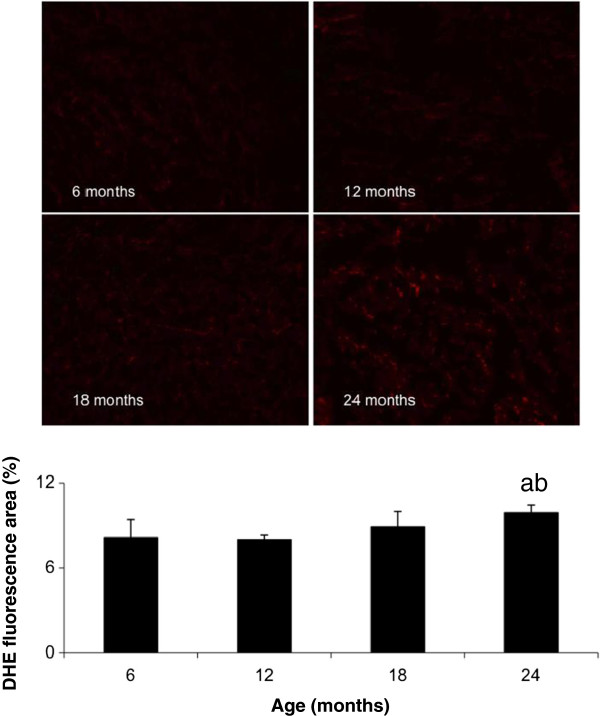
**O**_**2**_**‾ levels in aging skeletal muscle, by histological DHE examination, values represent the area of fluorescence, above a specified threshold, expressed as a percentage of the total area of the muscle cross-section*.*** The values represent the mean ± SEM. Statistically significant differences (P < 0.05) between groups are indicated by lower case letters, where a = 6 m/o, b = 12 m/o, c = 18 m/o and d = 24 m/o.

Ethidium fluorescence was elevated throughout the quadriceps muscle from 24 month old mice compared to 6 (*p* = 0.001) and 12 (*p* < 0.001) month old mice (Figure 2). Similar levels of fluorescence were observed between the other age groups (Figure 2).

### MRNA expression of Pro-oxidant and antioxidant enzymes in skeletal muscle of aging mice

The mRNA expression of the NOX enzyme subunits showed differential expression in skeletal muscle during aging. The catalytic NOX2 subunit was higher in the quadriceps muscle at 18 months compared to the younger 6 (*p* = 0.027) and 12 (*p* = 0.023) month old mice together with the older 24 (*p* = 0.047) month old mice (Table 2). Similarly, the mRNA expression of the NOX2 regulatory subunit, p40^*phox*^ was higher in the quadriceps muscle at 18 months compared to the younger 6 month old mice (*p* = 0.030, Table 2). Interestingly, the mRNA expression of the catalytic NOX2 type subunit p67^*phox*^ was higher in the quadriceps muscle at 24 months compared to the younger 6 (*p* = 0.007), 12 (*p* = 0.001) and 18 (*p* = 0.032) month old mice (Table 2). The mRNA expression of the NOX2 subunits, p22^*phox*^ and p47^*phox*^ and NOX2 subunit regulator, Rac1 were similar in the quadriceps muscle from all age groups. Like the NOX2 subunit itself, the mRNA expression of the catalytic NOX2 homologue, NOX4 was higher in the quadriceps muscle at 18 months compared to 6 months (*p* = 0.021, Table 2).

**Table 2:** Relative gene expression in aging skeletal muscle

	**Age (months)**			
	**6 (*n =*** **16)**	**12 (*n =*** **18)**	**18 (*n =*** **12)**	**24 (*n =*** **12)**
Gene expression (AU)				
NOX2	13.1 ± 2.9	11.0 ± 3.1	110 ± 14.7^abd^	23.0 ± 4.4
p22^*phox*^	17.8 ± 3.7	26.5 ±7.0	25.1 ± 9.4	48.7 ± 10.3
p40^*phox*^	14.0 ± 4.9	25.2 ± 8.7	57.7 ± 16.9^a^	42.9 ± 8.9
p47^*phox*^	3.1 ± 0.6	4.8 ± 1.4	16.4 ± 7.9	4.2 ± 1.2
p67^*phox*^	72.9 ± 13.4	41.1 ± 8.9	103 ± 33.0	272 ± 71^abc^
NOX4	1.5 ± 0.3	4.3 ± 1.0	9.2 ± 3.2^a^	5.1 ± 1.3
Rac1	1.2 ± 1.1	3.91 ± 0.9	2.9 ± 1.3	5.3 ± 0.8
SOD1	16.1 ± 5.2	19.3 ± 5.1	33.6 ± 11.0	69.6 ± 15.6^abc^
SOD2	7.9 ± 2.1	6.6 ± 1.5	6.7 ± 2.6	19.5 ± 4.0^abc^
SOD3	1.7 ± 0.4	1.51 ± 0.2	2.5 ± 1.3	2.9 ± 0.8
Gpx	16.5 ± 4.3	42.2 ± 10.2	56.4 ± 22.7	40.5 ± 6.0^a^
Catalase	19.4 ± 5.1	28.1 ± 8.1	34.4 ± 10.3	52.0 ± 9.0

The values represent the mean ± SEM, with all results normalised to 18 s ribosomal mRNA. Statistically significant differences (P < 0.05) between groups are indicated by lower case letters, where ^a^6 m/o, ^b^12 m/o, ^c^18 m/o and ^d^24 m/o.

The antioxidant enzymes also showed differential expression in skeletal muscle during aging. The mRNA expression of the cytosolic SOD1 and mitochondrial SOD2 antioxidant enzymes were higher in the quadriceps muscle at 24 months compared to the younger 6 (*p* = 0.005, *p* = 0.016), 12 (*p* = 0.010, *p* = 0.006) and 18 (*p* = 0.043, *p* = 0.012) month old mice (Table 2). Similarly, the mRNA expression of the antioxidant enzyme GPx was higher in the quadriceps muscle at 24 months compared to the younger 6 month old mice (*p* = 0.031, Table 2). There was no change in the mRNA expression of the extracellular SOD3 antioxidant enzyme and catalase in the quadriceps muscle from all age groups (Table 2).

### Protein levels of NOX2 enzyme subunits in skeletal muscle of aging mice

**Figure 3:**
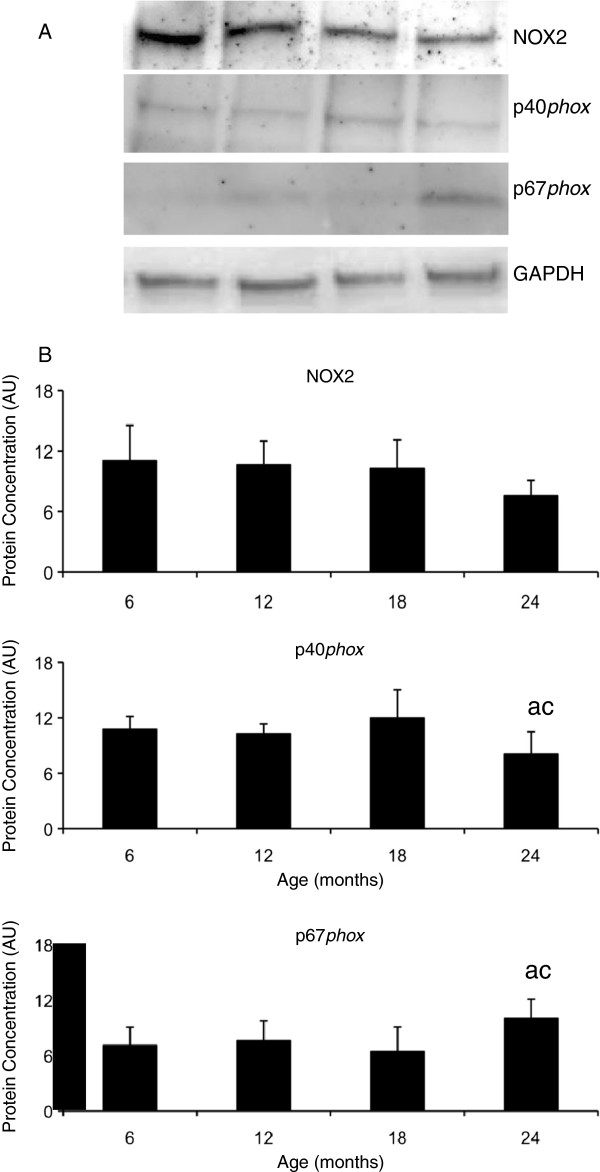
**The protein levels of the NOX2 enzyme subunits and protein levels in aging skeletal muscle*. *A**. Representative blots for NOX2, p40^*phox*^, p67^*phox*^ and GAPDH. **B**. Graphical representation of protein concentration expressed as arbitrary units (AU) after normalisation for GAPDH concentration. The values represent the mean ± SEM. Statistically significant differences (P < 0.05) between groups are indicated by lower case letters, where a = 6 m/o, b = 12 m/o, c = 18 m/o and d = 24 m/o.

Changes in the mRNA levels of the key NOX2 enzyme subunits; NOX2, p40^*phox*^ and p67^*phox*^ lead to further analysis of protein expression. The protein expression of the regulatory NOX2 enzyme subunit p40^*phox*^ was lower in the quadriceps muscle at 24 months compared to the younger 6 (p = 0.039) and 18 (p = 0.001) month old mice (Figure 3). However, the protein expression of the catalytic NOX2 enzyme subunit p67^*phox*^ was higher in the quadriceps muscle at 24 months compared to the younger 6 (p = 0.027) and 18 (p = 0.004) month old mice (Figure 3) and NOX2 protein expression was similar in all age groups (Figure 3).

### Antioxidant enzyme activity and hydrogen peroxide levels in skeletal muscle of aging mice

The changes in SOD mRNA expression lead to the investigation of SOD enzyme activity in skeletal muscle during aging. SOD1 activity levels were elevated in the quadriceps muscle at 18 months compared to the younger 6 (*p* < 0.001), 12 (*p* < 0.001) and older 24 (*p* = 0.011) month old mice (Table 3), and SOD1 activity in the quadriceps muscle at 12 months compared to 6 (*p* = 0.005), 18 (*p* < 0.001) and 24 (*p* < 0.001) months (Table 3). Additionally, SOD2 activity levels were lower in the quadriceps muscle at 24 months compared to the younger 6 (*p* < 0.001) and 12 (*p* < 0.001) month old mice (Table 3).

**Table 3:** Enzyme activity and hydrogen peroxide levels in aging skeletal muscle

	**Age (months)**			
	**6 (*n =*** **16)**	**12 (*n =*** **18)**	**18 (*n =*** **12)**	**24 (*n =*** **12)**
SOD1 (U/mg protein)	19.4 ± 1.3	9.1 ± 0.3^acd^	30.8 ± 2.5^abd^	25.9 ± 1.3
SOD2 (U/mg protein)	5.7 ± 0.5	5.7 ± 0.4	4.7 ± 0.3	3.4 ± 0.1^ab^
GPx (U/mg protein)	1.0 ± 0.2	1.1 ± 0.1^a^	0.7 ± 0.1^abd^	0.9 ± 0.2
Catalase (U/mg protein)	9.3 ± 0.9	12.1 ± 1.2^a^	8.8 ± 1.2^bd^	10.2 ± 0.9
Hydrogen Peroxide (μM)	1.12 ± 0.07	1.16 ± 0.04	2.42 ± 0.05^abd^	1.74 ± 0.06
Superoxide (AFU)	8.1 ± 1.3	8.0 ± 0.3	8.9 ± 1.1	10.0 ± 0.6^ab^

The values represent the mean ± SEM.

Statistically significant differences (P < 0.05) between groups are indicated by lower case letters, where ^a^6 m/o, ^b^12 m/o, ^c^18 m/o and ^d^24 m/o.

Changes in SOD antioxidant activity for the dismutation of O_2_‾ to H_2_O_2_ lead to further investigation of the H_2_O_2_ specific antioxidant enzymes, GPx and catalase. GPx activity levels were lower in skeletal muscle at 18 months compared to the younger 6 (*p* = 0.001), 12 (*p* < 0.001) and older 24 (*p* = 0.004) month old mice (Table 3). Similarly, catalase activity levels were lower in the quadriceps muscle at 18 months compared to the younger 12 (*p* < 0.001) and older 24 (*p* = 0.004) month old mice, and higher at 12 months compared to 6 months (*p* = 0.043) (Table 3).

Elevated levels of O_2_‾ and increased SOD activity lead to further investigations of H_2_O_2_ levels in skeletal muscle during aging. H_2_O_2_ were elevated in the quadriceps muscle at 18 months compared to the younger 6 (*p* < 0.001), 12 (*p* < 0.001) and older 24 (*p* = 0.002) month old mice (Table 3).

## Discussion

Sarcopenia has been described as a multifactorial syndrome developing from changes in muscle morphology, oxidative stress, inflammation, physical activity and nutrition [36]. The accumulation of ROS and evidence of oxidised proteins has been implicated in sarcopenic muscle, however the attenuation and suppression of this disease remains contradictory. The present study demonstrates a loss of skeletal muscle mass at 18 months that is indicative of sarcopenia. This sarcopenic muscle had elevated levels of H_2_O_2_ and low levels of the antioxidant enzymes, catalase and GPx. While the source of ROS in sarcopenic muscle remains relatively unknown, this study gives evidence for the NOX2 enzyme with an increase in the expression of the essential subunit components. This study also suggests a compensatory response to muscle insult with elevated levels of O_2_‾ and a decline in SOD1 protection and no further loss of muscle mass at 24 months.

The present study supports others who have demonstrated significant loss of skeletal muscle mass and cellular changes during aging [3,27,37]. Pansarasa et al. (1999) proposed that in humans, 65 years of age is the time of most significant change and associated damage in muscle [27]. Kimball et al. (2004) demonstrated age related decline in skeletal muscle mass at 18 months of age in mice and evidence of sarcopenia from 21-27 months of age [3]. Aging in general is associated with an increase in oxidative stress and steady rise over the lifespan [38]. Pansarasa et al. (1999) suggested a correlation between ROS activity and age related changes in human skeletal muscle [27], while a similar study by Mecocci et al. (1999) demonstrated age related oxidative damage and significant loss of muscle mass [39]. Studies to date, have investigated models of sarcopenia using young and old animals for muscle comparison [29,39-41], whereas the present study investigated aging skeletal muscle with four age groups that represent the progressive aging process and development of sarcopenia.

The significant decline in skeletal muscle mass found at 18 months was matched by a marked increase in the mRNA expression of NOX2 and p40^*phox*^, compared to 6 and 12 months. These two subunits are absolutely necessary for the regulation, assembly and activation of the NOX2 enzyme and generation of O_2_‾ [42]. The increase in NOX was not matched by an increase in O_2_‾, however further investigation in SOD1 activity suggests a well regulated antioxidant system in skeletal muscle at this time. The increase in O_2_‾ dismutation would indeed increase H_2_O_2_ levels in the cell and require antioxidant protection from catalase and GPx. However, despite the increase in H_2_O_2_ in skeletal muscle at 18 months compared to all other age groups, catalase and GPx appear to be unresponsive to the consequential increase in H_2_O_2_. The present study therefore gives evidence for antioxidant dysfunction during aging and at the onset of sarcopenia.

Increased levels of H_2_O_2_ have been found in aging skeletal muscle. Capel et al. (2004) demonstrated an increase in mitochondrial H_2_O_2_ release in the tibialis anterior (TA) muscles of 24 month old male Wister rats with sarcopenia when compared to 4.5 months [43], and this was later confirmed in the vastis lateralis muscle of elderly subjects [44]. Jackson et al. (2011) also demonstrated an increase in H_2_O_2_ levels in the gastrocnemius muscle of 18 and 24 month old mice when compared to the younger 6 months [45]. While Siu et al. (2008) found a similar increase in H_2_O_2_ levels in the gastrocnemius muscle of aged rats with significant loss of muscle mass, despite increased catalase activity [46]. Like these studies the present study supports H_2_O_2_ as a significant contributor in the development of sarcopenia.

A potential mechanism for H_2_O_2_, generated by NOX, in the development of sarcopenia has been proposed. Sriram et al. (2011) recently reported a feed forward loop of Myostatin (Mstn) induced TNF-α and NOX generated H_2_O_2_ as a second messenger where TNF-α in turn increases the expression of Mstn via transcription factor nuclear factor kappa beta (NF-κβ) [47]. Mstn is a known negative regulator of muscle growth. Overexpression of Mstn reduces muscle mass while Mstn deficiency has shown a 2 fold increase in muscle mass compared to wild types [48]. A recent study by McKay et al. (2012) demonstrated a 2 fold increase in myostatin mRNA levels in old muscle and suggests that myostatin impairs myogenic capacity in aged muscle [49]. In addition to the role of Mstn in sarcopenia, muscle wasting in sarcopenic rat muscle has been associated with increased Ubiquitin Proteasome System (UPS) activity [50]. Clavel et al. (2006) also reported increased UPS activity and expression of the atrophy related MuRF-1 and atrogin-1 genes in aged rat muscle that is suggested to be induced by TNF-α [51]. Furthermore, H_2_O_2_ has been shown to stimulate the UPS and expression of MuRF-1 and atrogin-1 in skeletal muscle myotubes [52]. Taken together, it is possible that NOX generated H_2_O_2_ acts as a second messenger for Mstn induced TNF-α and downstream regulation of the UPS in aging skeletal muscle sarcopenia.

However, H_2_O_2_ levels were not elevated in the quadriceps muscle at 24 months and there was no further loss of muscle mass. Instead, O_2_‾ levels were elevated in this muscle together with a decline in SOD1 activity. While this would suggest antioxidant dysfunction it is entirely possible that it is a regulated response to decrease H_2_O_2_ production and reduce further muscle wasting. Previous studies have demonstrated a compensatory mechanism in skeletal muscle that may occur in response to insult and the onset of muscle wasting. Edstrom et al. (2006) recently demonstrated a significant downregulation in atrophy related genes in sarcopenic skeletal muscle from 30 month old rats when compared to 4 and 12 months [53]. The potential decrease in muscle atrophy at 30 months could have been a compensatory response to earlier muscle loss that this particular study did not investigate. In further support of this, Kimball et al. (2004) found that while muscle mass began to decrease in rats beyond 21 months, markers of protein synthesis were elevated at 24 and 27 months of age [3]. A more recent study by Zhang et al. (2013), reported muscle weakness but no atrophy in 18 month old SOD1 deficient mice suggesting that SOD1 may be involved in muscle regenerative pathways [54]. Investigation into these complex systems in skeletal muscle would help to better understand the mechanisms involved in the development of sarcopenia.

The present study supports NOX2 enzyme involvement in the development of sarcopenia. The increase in the catalytic NOX2 enzyme subunit p67^*phox*^ in skeletal muscle at 24 months suggests that the source of O_2_‾ may be NOX2 generated. Like NOX2, NOX4 mRNA levels were also elevated in skeletal muscle at 18 months. In contrast to NOX2, NOX4 requires only p22^*phox*^ for oxidase activation and generation of O_2_‾ [9,55] and does not appear to reside in the plasma membrane, rather its localization is within intracellular organelles and generates O_2_‾ intracellularly [56,57]. However, the localisation of NOX4 in skeletal muscle is yet to be defined and therefore its role in skeletal muscle aging remains unknown. It is also important to note that because the NOX4 enzyme potentially generates O_2_‾ within subcellular compartments, such as the endoplasmic reticulum, it is undetectable by DHE [20,58]. Indeed, an increase in NOX4-generated O_2_‾ production in intracellular organelles has the potential to cause considerable damage to cellular structures and has been suggested to contribute to cellular senescence [20]. The results from this study indicate the potential for NOX4 as a subcellular regulator of O_2_‾ and suggests further investigation into its localization and thereby its role in skeletal muscle and sarcopenia.

## Conclusions

This study investigated oxidative systems in the development of sarcopenia in an otherwise healthy mouse model of aging. The time of significant change in skeletal muscle was at 18 months with an increase in NOX2 enzyme expression and levels of H_2_O_2_, antioxidant dysfunction and the onset of sarcopenia. However, while NOX2 enzyme expression was also increased in skeletal muscle at 24 months, O_2_‾ levels were elevated with SOD1 antioxidant dysfunction and maintenance of muscle mass. This study indeed supports oxidative changes in muscle during aging that may be both inductive of sarcopenia and a compensatory mechanism to maintain muscle integrity. This study suggests that elevated levels of H_2_O_2_ from NOX2 at 18 months, is a key component in the onset of sarcopenia. In contrast, the increase in O_2_‾ and decline in SOD1 activity at 24 months may be a regulated response to decrease H_2_O_2_ levels and further muscle insult. Furthermore, while this study suggests that H_2_O_2_ is the key oxidant in the onset of sarcopenia, the lack of antioxidant protection from catalase and GPx is equally as important, whereas the lack of SOD1 antioxidant protection at 24 months does not appear to contribute to sarcopenia.

## Competing interests

The authors declare that they have no competing interests.

## Authors’ contributions

MJS-G participated in the design of the study, carried out the analysis and interpretation of data and drafted the manuscript. PAL participated in the design of the study, contributed to the interpretation of data and revised the manuscript. Both authors read and approved the final manuscript.

## Pre-publication history

The pre-publication history for this paper can be accessed here:

http://www.biomedcentral.com/1471-2318/13/104/prepub
